# Symptomatology of Insanity
*Leçons Cliniques de Médecine Mentale. Par M. Falret. 8vo, pp. 270. Paris. 1854. Première partie. Symptomatologie Générale.


**Published:** 1854-10-01

**Authors:** 


### Art. IV.—SYMPTOMATOLOGY OF INSANITY.*

The Lectures, of which we propose to lay before our readers a brief analysis, were delivered by M. Falret, at the *Salpêtrière.* They were not originally intended for publication. Having, however, been reported in the *Gazette Médicale*, the author has, at the request of his pupils, consented to their separate publication. The author's far-spread reputation affords a strong presumption that the request was not made without good grounds. Our perusal of the volume has confirmed that opinion; and we would add, that by yielding to a reasonable solicitation, M. Falret has conceded a valuable contribution to the science of mental maladies.

The volume before us constitutes the first part of a series, and treats

* Leçons Cliniques de Médecine Mentale. Par M. Falret. 8vo, pp. 270. Paris. 1854. Première partie. Symptomatologie Générale.

of the *general symptomatology* of insanity; comprising lesions of sensation and propensities; of disorders of the intellect; of illusions and hallucinations; of derangements of the emotions and organic functions; with a general view of the course of insanity — *i. e.*, the successive evolution of its different phases.

These Lectures are preceded by an Introductory Discourse upon the general principles to be followed in the study of mental disorders. Four principal modes of observation, the author remarks, have been followed by those who, witnessing all the varieties of insanity, have learnt that there is no passion, no idea, which can arise in the human mind, which is not represented in a lunatic asylum. The difficulty of introducing the order and regularity of a scientific classification among elements so discordant as the multiplied and varied phenomena thus presented, has been made apparent by the marked differences in the mode of observation followed by different classes of observers. These are classed by the author under four principal groups—*e. g.*, those of the romancers, the narrators, the somatists, and the psychologists. The first were guided in their selection of phenomena by their singularity and *bizarrerie*, substituting pre-conceived ideas for exact observation of nature, fiction for reality. This is the mode followed in the infancy of art, when observation is abandoned to all the caprices of imagination and ill-directed curiosity. By the second mode, that of the narrators, or the merely descriptive, leaving the impulses of mere curiosity, a great step is taken in the way of true science, following the laws of induction, ascending from the particular to the general fact. This mode, which has prevailed until recently, has rendered, and will continue to render, great service to science. In searching for still greater perfection in the observation of the phenomena of insanity, two other directly contrary directions have been followed;—the somatic or physical, and the psychological. The somatic sees in all mental derangement a cerebral disorder, the symptoms of maladies various in their seat and nature: thus the incoherence of mania has been regarded and treated as identical with the delirium of fevers and other organic diseases. The psychologists, on the other hand, have too exclusively directed their attention to psychical phenomena, and have almost entirely neglected the physical conditions of organs:—thus, for instance, some regard insanity as consisting in lesions of attention, others as originating in moral causes. The regarding mental diseases too exclusively in either of these points of view, leads to artificial conclusions at variance with the actual phenomena presented in insanity. Thus very similar phenomena may originate in very different cause;—melancholy may proceed from stupidity, or from pre-occupa tion by some painful idea. The essential differences of mental disorders cannot be detected in any classification formed upon external and more obvious manifestations, the internal and less apparent conditions out of which they arise not having been duly studied.

In order to discover the tendencies, feelings, and dispositions which give rise to the manifestations of insanity, we must not be content merely to record the expressions and actions of the insane, as these individuals learn to conceal to a great extent their real mental condition. The first principle to be borne in mind, therefore, in the observation of the insane, is to change the part of the passive observer of phenomena into the active suggester of the manifestations of the fundamental condition. The second principle is to study the character of the individual patient, in order to distinguish the features he may present from those which are common to others. A third principle is never to separate a fact from the condition out of which it arises from the circumstances which precede or follow its occurrence. The physician who neglects the last principle resembles the historian who relates an historical event without investigating the causes which have prepared its way, and the circumstances of the epoch which may have modified or given it birth. A fourth principle, enjoined by M. Falret, consists in the establishment of *negative facts*, the discovery of the absence of conditions necessary to the sound mind, and by the observation of which the existence of insanity makes itself manifest more by the variance of the conduct of the insane in ordinary natures from those around them, rather than by their actual expressions.

In his second Lecture, M. Falret commences the more exact and immediate objects of his course — the symptomatology of mental diseases — by the consideration of derangements of sensibility and propensities in the insane. These the author presents in a twofold point of view—viz., their general and their special pathology, thereby having due regard to the laws of analogy, by collecting phenomena of a similar character, and the laws of difference, by describing the distinct characters of the separate facts. This method presents the difficulty of deciding whether a particular fact should find its place in a general or a special description; while it offers, also, the advantage that in a doubtful instance the case can be referred to general pathology, which should include all facts common to every species of insanity.

The author exposes the error, which he says is generally committed, of regarding lesions of sensibility, sentiment, propensity, &c., as isolated lesions; describing them as distinct affections, whereas they result from combined derangement of several faculties. Some writers, M. Falret remarks, have described insanity in the same way that the romancers have described the normal state. Thus, the history of a passion has become the history of a disease. They have depicted the religious, erotic, ambitious, homicidal, incendiary insane. Under this conviction, that the entire malady in its origin and its consequences is based upon an alteration of some one feeling or propensity, they have announced as distinct forms of monomania—*e.g.*, erotomania, demonomania, theomania, kleptomania, &c. In examining this doctrine, the author, in support of his views selects the sentiments of religion and of love, and the propensities to murder and to steal. When the words and acts of the insane religious mystic are closely examined, it will not be found that these proceed exclusively from an exaltation of religious principle, but are clearly referable to several different causes of a moral and intellectual order. Among these may be named pride and fear. The religious insane exhibiting temerity and audacity, believing themselves divinely inspired, are not simply content to make a few converts to their notions, but they would bring the whole world under the empire of their pride. Religion is but one of the manifestations of their sovereign pretensions; their words and their acts, properly interpreted, leave not the least doubt on this point, that these are far from exhibiting exalted religious feelings.

In a similar manner in the insane, who believe themselves to be the victims of the wrath of Heaven, damned, and devoted to the infernal powers, the fear of hell is but the expression of the general sentiment of fear. They think they perceive throughout the moral, and in the physical, world, all the evidences of ruin to themselves, and to their families and friends. What has been said with regard to religious insanity applies, the author observes, with more force to the passion of love. The erotic paroxysm is but a slight and passing ecstasy of an ideal love, with or without an object. The erotic form of insanity is very commonly associated with that of a religious character.

The requirements of legal medicine, M. Falret points out, have led to the attaching an undue importance to these propensities in regarding murder, suicide, theft, &c., as merely resulting from altered natural instincts, forgetting that either of these acts may proceed from very different sources. Some insane persons will commit murder to rid themselves of imaginary enemies, others to escape the power of some internal anxiety by which they are devoured; others will murder their children to send them to heaven. Other indications of insanity are also present in these cases. As in the healthy state, no faculty exists in an isolated state, so in the diseased condition the various faculties cannot be separated from each other.

Two forms of disordered sensibility are recognised by the author;—the state of depression and the state of exaltation. *Insanity without delirium does not exist;* that is to say, without any disturbance of the intellectual faculties. M. Falret observes, that insanity manifests itself variously, and that aberrations of sentiment are not less frequently met with than aberrations of ideas. Purely intellectual exertions induce a state of activity and tension not without their influence upon the state of sensibility; but these act rather as predisposing than as exciting causes of mental derangement, which is most frequently first perceived in disorders of the propensities or affections, as exhibited in aversion to those previously most warmly regarded. There is, in fact, an excess of susceptibility, inducing a want of harmony between the internal state and the world without; hence suspicion, defiance, melancholy, suicides, &c. These depressed conditions approximate to healthy states in which the mind is depressed, or under the influence of irritation, anger, sadness, &c. The opposite state of exalted sensibility has also its analogy in healthy conditions of the mind, in which its joy and happiness seem to extend to all around.

M. Falret notices two general forms of morbid change in the affections, one which consists in an exaggeration of previously existing dispositions, the other constituting an entire transformation of the personal character. Either of these may be insidious in its progress; but, however concealed, if once clearly detected, must be regarded as an indication of approaching insanity: this is especially true where the change is that of hatred for love towards those previously cherished, or of desire for things or persons hitherto shunned. In some cases the affections, instead of being either exaggerated or perverted are altogether annihilated; instead of an intelligent reasoning being, there remains only a mere automaton.

These disorders of the affections, M. Falret remarks, are always more concentrated, and are less apparent than disturbance of the intellectual faculties. The former are to be observed in the actions, while the latter reveals itself in the words. The moral attributes or affections are more closely allied to the conscience, the influence of which is necessary to self-government, and which does even occasionally control and oppose the manifestation of disorders of the affections. This authority not being equally put in force in the exercise of the intellectual faculties, the insane has no check upon the disorder of his ideas. Thus in melancholy it is more difficult to prove the existence of delirium than in mania.

The author reviews these various lesions of the affections under their relations to the two principal forms of mental diseases—general and partial insanity. In speaking of partial insanity, M. Falret observes that the predominance or persistence of certain sentiments has been singularly exaggerated. The insane having been represented as dominated exclusively and persistently by one clear and definite idea. Nothing can be more contrary to observation. Undoubtedly there is frequently in partial insanity the predominance of some one affection or propensity, or even of a particular series of ideas, neither however being exclusive, distinctly arranged, nor continuous. So far from of itself constituting the malady, the dominant sentiment or idea has generally been found to exist in the midst of a confused crowd of ideas; even in the least complex cases the patient is absorbed, not concentrated, in the sphere of a single affection. Moreover, the sentiment which is represented as continuous in its action, manifests itself, on the contrary, in a remittent manner, and as it were by paroxysms. The varieties of partial insanity in which the dominant lesion can be determined, are chiefly those which are denominated mania without delirium, moral insanity, and certain states of melancholy.

M. Falret proceeds to the consideration of disturbances of the intellectual powers in the insane. These he arranges under two general divisions, those characterized by torpor and those characterized by increased activity of the intellect. Torpidity or dulness is traced to two causes: the state of inertia of the intellectual faculties, and the preponderance of ideas or sentiments which engross the whole intellect, leaving it inaccessible to the external world and internal impressions, or, as we are wont to express it, the state of complete abstraction. These two conditions, although different, have often analogous modes of manifestation, which are to be guarded against in order to gain an exact idea of the malady, the treatment to be employed, and the opinion to be formed as to the prospect of cure. The apparent analogies are—penury of ideas, slowness of movements, and, moreover, the persistence of these two conditions despite diversity of excitement. The points of difference are to be found in the physiognomy: a certain degree of animation of countenance and concentration of traits, in the one case; a feebleness expressed in the features, and a vagueness in the eyes, in the other. In the latter also there is habitual weakness of memory, evidence of the feebleness of other faculties; in the former, under certain influences, the exclusive occupation with one train of thought can be diverted.

Increased activity of the intellectual faculties is equally remarkable in insanity: the abundance of ideas being greater than can be reduced to order, a confusion is produced and increased by the variety of emotions giving rise to them; so rapid and varied are the changes of expression and actions, that it requires to have witnessed it in order to the formation of any idea of the effect. In some cases this increased activity of the intellectual powers is not to be measured by the number of ideas, but by the predominance of certain faculties, as that of memory or imagination; for instance, the insane often excite astonishment by the recital of long passages from orators or poets, which had been supposed to have been long effaced from the memory; or they surprise by the prodigious facility with which they recal facts, dates, &c., with a precision of which previously they were incapable. The approach of a relapse into insanity is often correctly pre-indicated by an increased energy of intellectual faculties. It is to feebleness or other lesions of memory, combined with an exaltation of the feeling of wonder or self-love, and disorder of the judgment, that perverted notions of personality are to be traced.

M. Falret traces the disorder of the faculties of attention, judgment, imagination, will, conscience,—exhibited by the insane. Of the faculty of attention the author remarks, that incessantly scattered among or spread over an immense number of objects, it appears weaker than it really is, in general delirium; that in partial delirium it is often unemployed, the patient being completely absorbed; that in dementia the attention, like other faculties, is entirely abolished. The author here proceeds to combat the opinion of Esquirol, which attributes all disorders of the understanding to lesions of attention. This isolation of the faculties in the state of disease, as in health, appears to M. Falret to be arbitrary and impracticable. It is incorrect to attempt to resolve the disorders of all faculties into disorder of one only, overlooking the fact that all contribute or participate in different degrees.

Lesion of judgment, the author observes, is, without contradiction, the most prominent psychical phenomenon in mental alienation. "How could it be otherwise," he asks, "when to judge requires the concurrence, in the highest degree, of all the faculties." The judgment is, in fact, the exact expression of the rectitude or irregularity of all other faculties.

Regarding the free-will and conscience of the insane, M. Falret remarks, that the law is in accordance with science when it regards their lesion as the phenomenon in insanity the most constant and the most worthy of attention. That an insane person is not responsible for his acts is a principle held sacred in the legislation of all people. The author describes two profound modifications of the will, constituting distinct groups among the insane; the one in which it is absent, the other in which it is exalted. The former is more frequently observed in the outset of partial insanity with sadness, and has for manifestation extreme circumspection, very great indecision, and an inability to determine upon the most trivial circumstances. This feebleness of the will is also met with in more advanced stages of the disease, when the faculties show evidence of decay or complete ruin. The state of exaltation of will is denoted by excessive desires, and an imperious impulse to action; by a wish for the realization of every thought, for the execution of every plan, by a disposition to assume command; and generally by a tendency to delirium. These morbid states of the will are often derived from, and augmented by, lesions of sensibility.

From these considerations, the author continues, it is easy to infer that the insane have not a consciousness of good or evil in their acts; the three elements of our determinations, *savoir*, *vouloir*, and *pouvoir*, being at variance. The insane man is distinguished from the sane man by his exaggerated or illusory motives of action, by the suddenness of an impulse become imperious, and by the absence of the reflection which attends the acts of a reasonable being. The motives to any act are not wanting in the insane, but they are based upon illusion or hallucination: short of general delirium, or total obliteration of all faculties, conscience is not abolished in the insane, but their will and moral liberty are more or less strongly chained down or controlled in action by perversion of feeling or intellect, such as we see, in a minor degree, in passion.

As before observed, the author warns his hearers against the separation of the faculties in their disordered condition, and in the next place discusses the results of the morbid action of the faculties in the production of delirious ideas. The origin of delirious ideas in insanity is, by M. Falret, referred to two categories: they arise spontaneously, or are suggested by other ideas; by the same causes, in fact, which give rise to healthy ideas in the normal state. The former are the exceptional cases, and are indeed denied by those psychologists who deny the spontaneous origin of ideas in the mind.

For the production of a fixed predominant idea, both occasional or accidental causes, and permanent, profound, or predisposing causes, are requisite. In order that an idea shall take root, the soil must be prepared for its reception. The mind may hesitate long in making its selection before it may fix in a definite manner upon that which shall satisfy all the conditions into which it has been brought. "An attentive observer," remarks M. Falret, "tracing this first period of the evolution of a fixed idea, witnesses one of the most curious spectacles imaginable. He sees a man the prey of a disposition imposed by this malady, striving from time to time to rid himself thereof, but ever falling back under its tyrannical influence, and constrained by the laws of his mind to seek for some form under which to give it a body and a definite existence. He will be seen successively to adopt and to repel the divers ideas which present themselves to him, and laboriously striving to deliver himself of a delirium which shall be the expression, the exact image, of an internal condition of which himself, after all, suspects not the existence." This first phase in the evolution of the fixed idea, this gradual and progressive creation of delirium, constitutes the period of incubation of insanity.

Immediately that insanity is fully confirmed, a second distinct period in the development of the fixed idea is apparent. One principal idea forms the centre towards which other minor deliriums converge. Other delirious ideas he succeeds in systematizing in relation to the one fixed idea, constituting the truly acute period of mental disease. All accessory or secondary lesions of the intellect or sensibility gradually vanish, leaving the fixed idea stereotyped in the mind, and forming the chronic stage of the malady.

In the third Lecture, M. Falret discusses the nature of illusions in the insane, which he regards as in no way differing from other lesions of the intellectual faculties than that they have a sensation in place of an idea for their object. The author, however, treats of these separately, in obedience to custom. Esquirol had traced a marked difference between illusions and hallucinations; in the former there is a lesion of sense and an actual impression; in the latter a lesion of the brain and absence of external impression. In this distinction the author does not concur; he admits only one of these characters; he does not believe, at least in the majority of cases, in lesion of the sense in illusion, but he admits the existence of an external impression in the one case, and its absence in the other. M. Falret considers, contrary to Esquirol, that both illusion and hallucination are cerebral phenomena, of which the cause and the interpretation are to be found in lesions of the intellectual faculties. The difficulty, in practice, of detecting the lesion of the sense must not determine us to exclude altogether its influence in the production of illusion; at the same time it is maintained that this is very unfrequent, or at least is only occasionally the exciting cause of the intellectual lesion constituting the illusion.

In the fourth Lecture, the author compares certain physiological states with that of hallucination—such as dreams, somnambulism, &c. Hallucination, M. Falret designates a purely psychical symptom; one which denotes a transitory indisposition, or a disorder of the brain impending, or actually existing. It may, however, be entirely accidental, connected and disappearing with peculiar circumstances. Instances of this kind are given by the author; in these there has been an instantaneous production of images without belief in their reality, and consequently without delirium. Such hallucinations exist in periods of superstition and mysticism.

In the next place, the author proceeds to the consideration of hallucinations undoubtedly delirious, and to the solution of the question which has been answered in the affirmative by distinguished mental physicians, viz., the existence alone, as a form of insanity, of hallucinations confined to one sense. The author does not hesitate in his answer; as he does not admit the existence of madness limited to a single series of ideas, so he would reject the alleged monomania. The facts recorded in the annals of science, relating to hallucination of one sense, M. Falret asserts are very few, and those who have recorded them have overlooked their true relations. The hallucinations of distinguished men, by which the world has often been misled into a belief of their inspiration or intercourse with supernatural agencies, have been but the culmination of their delirium, which may have been so evanescent as to have escaped observation.

While denying the existence of sensorial monomania, the author recognises a form of insanity restricted to a small number of objects, and with predominant hallucination. As in ordinary partial insanity, it may be gay or sad, expansive or concentrated. In such cases the hallucination is the principal fact, the centre upon which turns the greater part of the intellectual and moral perversities. These hallucinations may present degrees of intensity, as they are not always equally clear and distinct in the persons in whom they exist. Thus, as to hallucinations of the sense of hearing, there will be at first merely buzzing and other noises in the ears, which become confused and mistaken for sounds of clocks, &c. Carried to a higher degree, the same are regarded as sounds from heaven, celestial harmonies, songs of birds, spiritual conversations. These gradations mark the phases of insanity in its commencement and also in its decline, when an hallucination will survive the delirium, and is no longer believed to be real by the patient. It is to be borne in mind, however, that the persistence of this phenomenon is to be regarded as a lingering spark, which may at any moment burst out again in a destructive conflagration.

In the following, the fifth Lecture, the author points out some of those characters of gesture, expression, &c., of the insane, which denote the presence of hallucinations. These, like illusions, lose their intensity and frequency in proportion as the intelligence diminishes, *i. e.*, in the chronic state, or dementia. Hallucinations presuppose too great activity of the mind, too exalted a condition of the imagination, to be compatible with idiotcy.

The degree of mental cultivation, or of development of the intellectual powers, more particularly of the imagination, produces great difference, not in the fundamental nature of this psychical phenomenon, but in its intensity, and the number and variety of its images. Where the mind has been but little cultivated, the delusions are usually connected with the most ordinary affairs of life; in the more highly accomplished, the dreams and hallucinations often form *tableaux* possessing both beauty and grandeur.

M. Falret examines the statement of Esquirol, that of one hundred insane individuals, forty-five at least have hallucinations. This estimate, although generally admitted, is not borne out by the author's observations. Of one hundred and three patients of all ages, in the Salpêtrière, presenting all varieties of insanity, under the author's care for various periods, from fourteen days to five years, averaging eighteen months, only thirty-two were found to possess hallucinations, either simple or complex. Of one hundred and ten inmates of the establishment at Vannes, of both sexes, at all ages and varieties of mental disease, only thirty-four presented hallucinations. The causes of the exaggeration of the numbers of insane having hallucinations are, in the first place, the difficulty of distinguishing between these and illusions, and the different interpretations put upon the language of the insane. A second cause of error is to be found in the circumstance that the insane makes himself the centre of all occurrences surrounding him, by which means he interprets everything according to his own distorted views, and transforms unimportant occurrences into events of the highest importance.

From the consideration of hallucinations in general, the author proceeds to the consideration of these as manifested in the special senses.

In the sixth Lecture, the author examines the principal theories that have been propounded for the explanation of hallucinations, refuting both the theories which consider these to have a sensorial origin and nature, and those which regard them as essentially intellectual, or partly intellectual and partly sensorial. In M. Falret's opinion, hallucinations can be explained only by regarding them as lesions of the imagination, not, however, entirely excluding all cerebral influence; that hallucinations belong to a modification of the cerebral action analogous to that which in the healthy state attends the exercise of the imagination. The imagination acts without control in dreams, and produces hallucinations; in the waking state also the imagination, operating upon the materials furnished by the memory, creates veritable images, varying with individual disposition, but which are almost uniformly at variance with reality. The author sums up his observations on this subject in the following manner:—

"1. That Esquirol has based his distinction, between hallucination and illusion, upon two secondary characters; a lesion of sensation and the actuality of the impression in the one case, and upon the absence of these in the other.

"2. That in order to establish these differences truly scientifically, they must be sought for in the domain of the intellect.

"3. That the facts collected by Esquirol, under the name of illusions, should be divided in two categories, in one of which these are almost identical with hallucinations, while in the other they differ entirely, and are confounded with other phenomena of delirium."

The seventh Lecture treats of the physical phenomena of insanity. These are arranged under the three several heads of lesions of sensation, motion, and organic functions. Lesions of sensibility are divided into the general and the partial, these two states affording grounds of the distinction of the disease into the expansive and the depressive forms. Disordered conditions of the general sensibility lead patients to believe that certain parts of their bodies have undergone change of shape or proportion, or that the composition of their entire frame has undergone metamorphosis. Phenomena of a similar nature are known to occur in ordinary dreams. Diminution of sensibility is less frequently met with than its exaltation. In many patients delusions depend upon perverted sensation. In others there is an entire indifference to suffering, under which state they will inflict mutilations, &c., expose themselves to frost, snow, rain, &c., without manifesting indications of pain. These, however, as the author observes, are exceptional cases, an indifference to changes of weather not being an invariable attendant upon insanity.

Lesions of motion demand attention, as they may be symptoms of cerebral disease, as the bases of prognosis, and as the source of indications of treatment. The various modifications and derangements of the muscular system, as expressed in the countenance and in the limbs, and as exhibited in chorea, spasms, convulsions, &c., are glanced at by the author in relation to mental maladies. Among the lesions of the organic functions noticed by M. Falret as occurring in the insane, are sleep, generation, nutrition, secretion, pulsation, respiration.

In the three following Lectures, M. Falret points out the course of insanity, describing the signs of predisposition, the period of incubation and gradual evolution, the period of invasion, and the actual existence of the disease; its various phases, changes, complications, and terminations.

These latter pages of the author's work are so strictly practical, that to have done them justice we must have transferred them to our pages; but as, in reality, their value arises out of the truthfulness with which well-known phenomena are depicted and set before the reader, we shall better discharge our trust by referring our readers to the treatise wherein they are contained.

